# SARS-CoV-2 Exposure in Escaped Mink, Utah, USA

**DOI:** 10.3201/eid2703.204444

**Published:** 2021-03

**Authors:** Susan A. Shriner, Jeremy W. Ellis, J. Jeffrey Root, Annette Roug, Scott R. Stopak, Gerald W. Wiscomb, Jared R. Zierenberg, Hon S. Ip, Mia K. Torchetti, Thomas J. DeLiberto

**Affiliations:** US Department of Agriculture (USDA) National Wildlife Research Center, Fort Collins, Colorado, USA (S.A. Shriner, J.W. Ellis, J.J. Root, T.J. DeLiberto);; Utah Division of Wildlife Resources, Salt Lake City, Utah, USA (A. Roug);; USDA Wildlife Services, Boise, Idaho, USA (S.R. Stopak);; USDA Wildlife Services, Billings, Montana, USA (G.W. Wiscomb); U; SDA Wildlife Services, Salt Lake City (J.R. Zierenberg);; US Geological Survey National Wildlife Health Center, Madison, Wisconsin, USA (H.S. Ip);; USDA Veterinary Services, Ames, Iowa, USA (M.K. Torchetti)

**Keywords:** American mink, *Neovison vison*, fur, farm, rodents, outbreak, spillover, SARS-CoV-2, COVID-19, coronavirus disease, severe acute respiratory syndrome coronavirus 2, viruses, respiratory infections, zoonoses

## Abstract

In August 2020, outbreaks of coronavirus disease were confirmed on mink farms in Utah, USA. We surveyed mammals captured on and around farms for evidence of infection or exposure. Free-ranging mink, presumed domestic escapees, exhibited high antibody titers, suggesting a potential severe acute respiratory syndrome coronavirus 2 transmission pathway to native wildlife.

We report a wildlife epidemiologic investigation of mammals captured on or near properties in Utah, USA, where outbreaks of severe acute respiratory syndrome coronavirus 2 (SARS-CoV-2) infection occurred in farmed mink. Mink farms are relatively common in the United States, and most are small family farms. The US Department of Agriculture’s National Veterinary Services Laboratories (Ames, IA, USA) confirmed SARS-CoV-2 in mink at 2 Utah farms on August 17, 2020, after an investigation by the Utah Veterinary Diagnostic Laboratory and the Washington Animal Disease Diagnostic Laboratory (*1*). SARS-CoV-2 outbreaks have subsequently been confirmed at multiple farms in Utah, Michigan, Wisconsin, and Oregon. Although epidemiologic investigations are ongoing, infected workers are the probable source of the virus’s introduction (*2*).

The first reported SARS-CoV-2 infection in mink occurred in the Netherlands in April 2020 (*3*). Since then, dozens of farms in Europe have experienced outbreaks, and more than a million mink have been culled (*2*). Genetic analyses suggest spillover from human infections, and potential zoonotic transmission from mink to a worker is suspected (*4*). Clinical data from mink infected with SARS-CoV-2 indicate the species is highly susceptible and that infections can range from asymptomatic to peracute (*5*).

We captured free-roaming mammals during August 22–30, 2020, by using Sherman (rodents) and Tomahawk (mesocarnivores) traps placed outside of barns and barrier fences on outbreak premises and public lands within a 3.5-km buffer zone. Sample collection included oral, nasal (washes for mice), and rectal swab specimens; tissue specimens; and blood specimens. Swabs were placed in cryovials filled with 0.5 mL viral transport medium, and tissue specimens were placed in plastic sample bags or cryovials. Serum and swab samples were stored on ice, and tissue specimens were flash frozen on dry ice. Most samples were shipped within 24 hours. Swabs and serum samples were shipped to the National Veterinary Services Laboratories, and tissue specimens were sent to the US Geological Survey National Wildlife Health Center (Madison, WI, USA) for testing. All swabs and tissue specimens were tested for SARS-CoV-2 viral RNA by real-time reverse transcription PCR (rRT-PCR) targeting the N1 and N2 genes, and serum samples were tested by virus neutralization assay (*6*). A positive rRT-PCR result was defined as detection of both N1 and N2.

We captured 102 mammals (78 rodents and 24 mesocarnivores). Rodent captures consisted of 45 deer mice (*Peromyscus maniculatus*), 5 *Peromyscus* spp. mice, 25 house mice (*Mus musculus*), and 3 rock squirrels (*Otospermophilus variegatus*). Mesocarnivore captures consisted of 11 presumed escaped American mink (*Neovison vison*), 2 presumed wild American mink, 5 raccoons (*Procyon lotor*), and 6 striped skunks (*Mephitis mephitis*). Presumed escaped mink were closely associated with barns and designated as domestic escapees on the basis of location, behavior, and appearance. We identified wild mink by brown coat color and smaller size compared with farmed mink. All escaped mink and rodents, except for 4 deer mice and 1 rock squirrel, were caught on farm premises. All raccoons, the 2 presumed wild mink, and all but 1 striped skunk were captured off-property but within the buffer zone.

Serum samples from the 11 mink escapees tested positive for SARS-CoV-2 antibodies by virus neutralization (Table). No other animal had a detectable antibody response. Of the antibody-positive escaped mink, 3 also had high cycle threshold (C_t_) detections by rRT-PCR of nasal swabs (range C_t_ 35.89–38.95) and 1 lung tissue specimen (C_t_ 39.2 for N1). A rectal swab specimen from a house mouse had a high C_t_ detection by rRT-PCR but was negative for SARS-CoV-2 antibodies. N1 alone was detected by rRT-PCR in 2 samples from deer mice (C_t_ 37.55 and 39.57).

**Table:** Virus neutralization titers and rRT-PCR cycle thresholds for samples collected from captured mammals in study of SARS-CoV-2 exposure in escaped mink, Utah, USA, August 2020

Animal ID	Animal	Capture date	Site	Virus neutralization titer	rRT-PCR C_t_†	rRT-PCR sample type
ID000003	Escaped mink	2020 Aug 23	A	128	ND	ND
ID000004	Escaped mink	2020 Aug 23	A	128	ND	ND
ID000101	Escaped mink	2020 Aug 24	A	512	ND	ND
ID000010	Escaped mink	2020 Aug 24	A	512	38.80	Nasal swab
ID000011	Escaped mink	2020 Aug 24	A	512	ND	ND
ID000012	Escaped mink	2020 Aug 24	A	512	38.95	Nasal swab
ID000013	Escaped mink	2020 Aug 24	A	128	ND	ND
ID000014	Escaped mink	2020 Aug 24	A	256	‡	Nasal swab, lung tissue
ID000052	Escaped mink	2020 Aug 30	B	64	‡	Nasal swab
ID000053	Escaped mink	2020 Aug 30	B	256	37.38	Nasal swab
ID000054	Escaped mink	2020 Aug 30	B	128	ND	ND
ID000030	Feral or wild mink	2020 Aug 26	C	Negative	ND	ND
ID000051	Feral or wild mink	2020 Aug 28	C	Negative	ND	ND
ID000017	House mouse	2020 Aug 25	C	Negative	35.89	Rectal swab
ID000064	Deer mouse	2020 Aug 30	B	Negative	‡	Oral swab
ID000093	Deer mouse	2020 Aug 31	B	Negative	‡	Rectal swab

*C_t_, cycle threshold; rRT-PCR, real-time reverse transcription PCR; SARS-CoV-2, severe acute respiratory syndrome coronavirus 2; ND, not detected.  †Mean C_t_ across the N1- and N2-genes; a positive result is defined as detection of both N1 and N2. ‡C_t_ detection of N1-gene only.

Experimental studies of rodents suggest that Old World rodents (e.g., *Mus*) are resistant to SARS-CoV-2 infection (*7*) and New World species (e.g., *Peromyscus*) are susceptible (A. Fagre, unpub. data, https://doi.org/10.1101/2020.08.07.241810; B.D. Griffin, unpub. data, https://doi.org/10.1101/2020.07.25.221291). Given the demonstrated resistance of house mice to SARS-CoV-2 infection, the high-C_t_ rectal swab specimen, in absence of other positive results, suggests potential ingestion and excretion of contaminated material (e.g., food, carcasses) rather than infection. In contrast, the antibody responses of all escaped mink combined with high-C_t_ swab specimens for some animals suggests recent SARS-CoV-2 infections. These exposures in escaped mink are unsurprising given biosecurity practices on some premises did not exclude incursions of escaped mink into barns.

Although we did not find evidence for SARS-CoV-2 establishment in wildlife, the discovery of escaped mink with the opportunity to disperse and interact with susceptible wildlife, such as wild mink or deer mice, is concerning. In Utah, mink farms often overlap with designated critical mink habitats. Interactions or shared resources between escaped mink and wild mink or other wildlife species represent potential transmission pathways for spillover of SARS-CoV-2 into wildlife and could lead to health consequences or establishment of new reservoirs in susceptible wildlife (*8*; A. Fagre, unpub. data, https://doi.org/10.1101/2020.08.07.241810). Heightened biosecurity and best management practices would help prevent accidental releases of infected animals or spillover of SARS-CoV-2 from susceptible species to native wildlife.
